# Mutagenic Analysis of the Putative ABCC6 Substrate-Binding Cavity Using a New Homology Model

**DOI:** 10.3390/ijms22136910

**Published:** 2021-06-27

**Authors:** Flora Szeri, Valentina Corradi, Fatemeh Niaziorimi, Sylvia Donnelly, Gwenaëlle Conseil, Susan P. C. Cole, D. Peter Tieleman, Koen van de Wetering

**Affiliations:** 1 Department of Dermatology and Cutaneous Biology and PXE Center of Excellence in Research and Clinical Care, Thomas Jefferson University, Philadelphia, PA 19107, USA; szeri.flora@ttk.hu (F.S.); Fatemeh.Niaziorimi@jefferson.edu (F.N.); Sylvia.Donnelly@jefferson.edu (S.D.); 2 Research Centre for Natural Sciences, Institute of Enzymology, 1117 Budapest, Hungary; 3 Department of Biological Sciences and Centre for Molecular Simulation, University of Calgary, Calgary, AB T2N 1N4, Canada; vcorradi@ucalgary.ca (V.C.); tieleman@ucalgary.ca (D.P.T.); 4 Department of Pathology and Molecular Medicine, Queen’s University, Kingston, ON K7L 3N6, Canada; conseilg@queensu.ca (G.C.); spc.cole@queensu.ca (S.P.C.C.)

**Keywords:** ABC transporter, pseudoxanthoma elasticum, homology modeling, substrate-binding site, cellular ATP efflux, mutagenesis

## Abstract

Inactivating mutations in ABCC6 underlie the rare hereditary mineralization disorder pseudoxanthoma elasticum. ABCC6 is an ATP-binding cassette (ABC) integral membrane protein that mediates the release of ATP from hepatocytes into the bloodstream. The released ATP is extracellularly converted into pyrophosphate, a key mineralization inhibitor. Although ABCC6 is firmly linked to cellular ATP release, the molecular details of ABCC6-mediated ATP release remain elusive. Most of the currently available data support the hypothesis that ABCC6 is an ATP-dependent ATP efflux pump, an un-precedented function for an ABC transporter. This hypothesis implies the presence of an ATP-binding site in the substrate-binding cavity of ABCC6. We performed an extensive mutagenesis study using a new homology model based on recently published structures of its close homolog, bovine Abcc1, to characterize the substrate-binding cavity of ABCC6. Leukotriene C4 (LTC_4_), is a high-affinity substrate of ABCC1. We mutagenized fourteen amino acid residues in the rat ortholog of ABCC6, rAbcc6, that corresponded to the residues in ABCC1 found in the LTC_4_ binding cavity. Our functional characterization revealed that most of the amino acids in rAbcc6 corresponding to those found in the LTC_4_ binding pocket in bovine Abcc1 are not critical for ATP efflux. We conclude that the putative ATP binding site in the substrate-binding cavity of ABCC6/rAbcc6 is distinct from the bovine Abcc1 LTC_4_-binding site.

## 1. Introduction

Inactivating mutations in the gene encoding ATP-binding cassette (ABC) subfamily C member 6 (ABCC6) underlie the autosomal recessive disease pseudoxanthoma elasticum (PXE, OMIM #264800) [1,2,3], characterized by ectopic mineralization in the skin, eyes, and vascular system [4,5,6]. PXE is a slowly progressing connective tissue disorder that affects approximately 1 in 50,000 individuals worldwide [7]. There is currently no specific and effective therapy for PXE and the disease slowly progresses after initial diagnosis [8].

ABCC6 is predominantly expressed in the liver [9] where it mediates the release of ATP from hepatocytes into the bloodstream [10,11]. Outside the hepatocytes, yet still in the liver niche, the released ATP is converted into AMP and the mineralization inhibitor pyrophosphate (PPi), by ectonucleotide pyrophosphatase phosphodiesterase 1 (ENPP1) [12]. The absence of ABCC6-mediated ATP release in both PXE patients and Abcc6 null mice results in plasma PPi levels that are < 40% of those found in ABCC6-proficient individuals [11], providing a plausible biochemical explanation for their ectopic mineralization. Moreover, plasma PPi levels decline during pregnancy, which might explain the increased risk of vascular calcification in multiparous individuals [13]. Recent data indicate that ATP efflux by the progressive ankylosis protein (ANK) is also a major determinant of plasma PPi levels [14]. Intriguingly, an ABC protein other than ABCC6 has been reported to also be involved in cellular ATP release, albeit indirectly, as two ABCG1 variants were found to control volume-regulated anion channel-dependent ATP release by regulating cholesterol levels in the plasma membrane [15]. Neither of these ABCG1 variants, however, have been implicated in the pathology of PXE.

Although low levels of circulating PPi explain why PXE patients suffer from ectopic mineralization, the molecular details of ABCC6-mediated ATP release remain elusive. Most ABC proteins of the C-branch function as ATP-dependent efflux transporters, though there are several exceptions. Thus, ABCC7 is the ATP-gated chloride channel cystic fibrosis transmembrane conductance regulator (CFTR) with inactivating mutations causing cystic fibrosis [16], and ABCC8 and ABCC9 are regulatory subunits of complex potassium channels [17].

Most of the currently available data indicate that ABCC6 is an ATP-dependent ATP efflux transporter: ATP efflux rates from ABCC6-transfected HEK293 cells are very similar to rates at which ABCC1, ABCC2, and ABCC3 transport morphine-3-glucuronide out of cells [18]. Moreover, our recent work indicates ABCC6 does not function as an ATP channel [19] and nor does it induce the exocytosis of ATP-loaded vesicles (our unpublished data). ABCC6 was initially implicated in the transport of glutathione conjugates in in vitro vesicular uptake assays [20,21] but these results proved difficult to reproduce in later studies [6].

In 2017, the structure of LTC_4_-bound bovine Abcc1 (bAbcc1) in the ATP-free state, with a bipartite transmembrane cavity open towards the cytosol (inward-facing) was reported using cryogenic electron microscopy (cryoEM) [22]. This report was later followed by the cryoEM structure of the ATP-bound, outward-facing state of bAbcc1, with the transmembrane cavity open to the opposite side of the membrane [23]. Given that (1) ABCC6 shares most sequence similarity with ABCC1 [24], (2) the genes encoding both proteins arose from a recent gene duplication [25], and (3) in vitro studies suggested both proteins might share LTC_4_ as a substrate [4,21,26,27] though attempts to connect the transport of LTC_4_ to the potential role of ABCC6 failed [6].

We used the ATP-free, LTC_4_-bound and ATP-bound, substrate-free, bAbcc1 cryoEM structures as templates to build inward- and outward-facing homology models of hABCC6 and rat Abcc6 (rAbcc6) as a means of identifying amino acids potentially forming the binding cavity for ATP. Amino acids in ABCC6 at the same positions as those in bAbcc1 comprising the proposed bipartite binding cavity of LTC_4_ were subsequently mutated in rAbcc6 expression vectors and the mutant rAbcc6 proteins functionally characterized to determine if they play a role in ABCC6-dependent ATP release. Several of the introduced mutations did not markedly alter rAbcc6 activity and thus are not essential for ATP efflux. Strikingly, the generation of a rAbcc6 mutant in which all amino acids of the modeled binding cavity were changed into their ABCC1 counterparts, showed ATP efflux similar to the wild-type protein.

## 2. Results

### 2.1. Homology Models of Human and Rat ABCC6/Abcc6

ABC transporters have a common structural core that in ABCC6 and all other ABCC subfamily members consist of transmembrane domain 1 (TMD1, encompassing transmembrane helices (TM) 6 to 11), nucleotide-binding domain 1 (NBD1), TMD2 (TM12 to 17) and NBD2. We built a sequence alignment for TMD1-NBD1 and TMD2-NBD2 (see Supporting Information files TMD1-NBD1_Alignment.pdf and TMD2-NBD2_Alignment.pdf), using several orthologues of ABCC1, ABCC6, and ABCC5, for which negatively charged substrates have been reported. From these alignments, we observed a 46% and 48% sequence identity for rAbcc6-bAbcc1 and hABCC6-bAbcc1 TMD1-NBD1, respectively, and 52% and 53% sequence identities for rAbcc6-bAbcc1 and hABCC6-bAbcc1 TMD2-NBD2, respectively. The % sequence identity calculated for TMD1 (rAbcc6 residues 298–608) and TMD2 (rAbcc6 residues 933–1242) are 39% and 48%, respectively, for rAbcc6 and bAbcc1, and 40% for TMD1 and 48% for TMD2 between hABCC6 and bAbcc1. Considering the high sequence identity with bAbcc1, we modeled the structural core for rAbcc6 and hABCC6 in two distinct conformational states (Figure S1), based on the cryoEM structures reported for the LTC_4_-bound, ATP-free, inward-facing state and on the ATP-bound outward-facing state of bAbcc1 [22,23].

**Figure 1:**
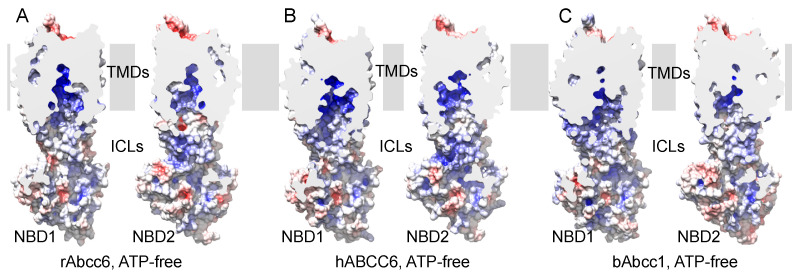
Electrostatic potential of the inward-facing state of hABCC6/rAbcc6 and bAbcc1. Electrostatic potential mapped on the molecular surface of the ATP-free, inward-facing (**A**) rAbcc6 model, (**B**) hABCC6 model, and (**C**) bAbcc1 cryoEM structure. The isovalue was set at −10 k_B_T/e for the negative potential (red) and +10 k_B_T/e for the positive potential (blue). For each transporter, the surface is clipped, and the two halves are shown side by side. The region of the transporters embedded in the membrane is highlighted by the gray slab. TMDs, transmembrane domains; ICLs, intracellular loops, i.e., the intracellular extension of the TMDs; NBD1 and NBD2, nucleotide binding domain 1 and 2.

Consistent with their relatively high degree of sequence identity, both hABCC6/rAbcc6 and bAbcc1 showed a strong positive potential in the cavity along TMs of both TMD1 and TMD2 (Figure 1). Another common feature of hABCC6/rAbcc6 and bAbcc1 is the presence of a more negative potential on the extracellular end of the TMDs (Figure S2A), which following the conformational change to the ATP-bound, outward-facing state, appears less prominent (Figure S2).

**Figure 2:**
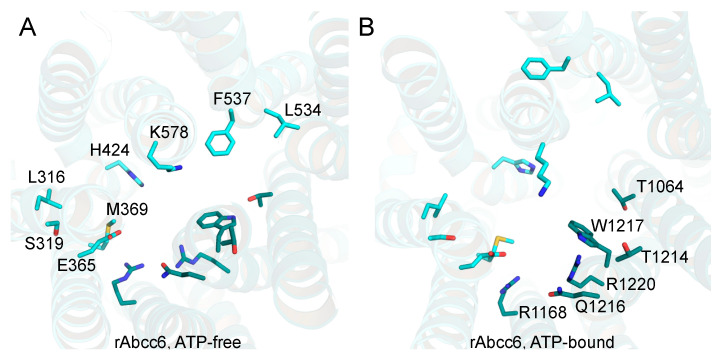
rAbcc6 residues in the transmembrane cavity corresponding to those in the bAbcc1 cavity surrounding LTC_4_. View from the extracellular side of the transmembrane cavity of the inward-facing, ATP-free (**A**) and outward-facing, ATP-bound (**B**) models of rAbcc6. The residues corresponding to those of the bAbcc1 LTC_4_ binding cavity are shown as sticks in light cyan for TMD1 and in teal for TMD2.

In the transmembrane cavity of bAbcc1, several residues have been proposed as participating in the recognition of LTC_4_ through a network of hydrogen bonds, salt bridges, and van der Waals contacts (Figure S3, Figure 2 and Table 1) [22], although not all of the proposed interactions are supported by biochemical studies [28,29].

The residues in the cavity are proposed to form a bipartite binding site [30] with a more prominent positive charge on one side (residues K332, H335, L381, F385, Y440, F594 of TMD1, and R1196, N1244, and R1248 of TMD2, namely the P-pocket) to bind the hydrophilic glutathione moiety of LTC_4_ and a more hydrophobic pocket (namely, the H-pocket) to accommodate the lipid tail of LTC_4_ (residues T550, W553 of TMD1, and M1092, Y1242, W1245 of TMD2) [22]. Our goal was to address if ABCC6 (the corresponding residues shown in Figure 2 for rAbcc6 and in Figure S3 for hABCC6), in both the ATP-free and ATP-bound states, might be involved in ATP interaction and efflux. First, we analyzed how the proposed P-pocket and H-pocket residues are conserved across the sequences taken into account to build the models. The analysis of the sequence alignment (Supplemental information files TMD1-NBD1_Alignment.pdf and TMD2-NBD2_Alignment.pdf) showed that bAbcc1 W553 and W1245 in the hydrophobic pocket are conserved among the ABCC6, ABCC1, and ABCC5 sequences, and correspond to rAbcc6 residues F537 (TM10 in TMD1) and W1217 (TM17 in TMD2) (Figure 2). In the P-pocket, the more conserved residues are bAbcc1 R1196, R1248, and N1244, which correspond to rAbcc6 R1168 (TM16 in TMD2), R1220 and Q1216 (TM17 in TMD2) (Figure 2).

The degrees of similarity for the other residues of the bAbcc1 P- and H-pockets vary across the ABCC1, ABCC6, and ABCC5 sequences considered in the alignment. Charged residues that are not conserved are: (1) bAbcc1 K332, which is a leucine in ABCC6 (L316 in rAbcc6) and ABCC5, (2) H335 in hABCC1/bAbcc1, which is a serine in the hABCC6/rAbcc6 sequences, and (3) rAbcc6 E365, which is a glutamate only in ABCC6 sequences and a leucine in hABCC1 (L381 in bAbcc1) and ABCC5. Of note, in hABCC1, K332, and to a lesser extent H335, are indispensable for LTC_4_ binding and transport, indicating K332 and H335 are crucial amino acid residues in its LTC_4_-binding site [29,31]. An additional alignment performed using sequences of human ABC transporters of the C subfamily confirmed these observations and demonstrate exceptionally high conservation of R1168 and R1220 (numbering of rAbcc6) among ABCC proteins (Figure S5).

### 2.2. Functional Analysis of Single Amino Acid rAbcc6 Mutants

**Figure 3:**
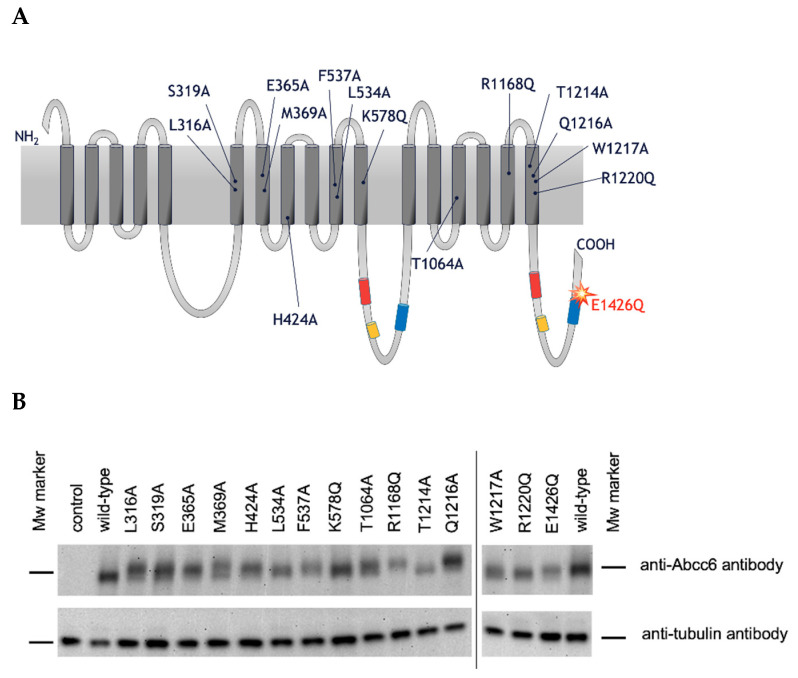
(**A**): Topology of the rAbcc6 amino acids analogous to those that comprise the LTC_4_ binding cavity in bAbcc1. The inactivating mutation in the NBD2, E1426Q, is also indicated (**B**): Expression of the rAbcc6 single amino acid mutants in HEK293 cells. Of the total cell protein, 5 µg was fractionated on a 7.5%-polyacrylamide gel and bands corresponding to wild-type and mutant rAbcc6 proteins and the housekeeping protein tubulin were detected by Western-blot analysis using the K14 anti-rat Abcc6 antibody and the anti-tubulin antibody, respectively. The slight differences in electrophoretic mobility of some of the mutants may be attributed to altered glycosylation or other post-translational modifications.

Our aim was to determine whether the residues in ABCC6 corresponding to those thought to be important in interaction with the physiological ABCC1 substrate LTC_4_, play a role in ABCC6-mediated ATP efflux. We used rAbcc6 in these studies, because it has higher activity in HEK293 cells than hABCC6 [10]. ATP and other nucleoside triphosphates (NTPs), the putative physiological substrates of ABCC6, carry multiple negative charges. We hypothesized such negatively charged substrates may be “coordinated” by positively charged residues in the substrate binding cavity of rAbcc6. Therefore, positively charged amino acid moieties (i.e., lysine, arginine, and histidine) at these positions, were replaced with uncharged residues (i.e., glutamine and alanine). Non-charged amino acid residues, according to the canonical/conservative mutagenesis practices, were changed into cavity-creating alanine residues, aimed at retaining the overall structure of the protein. The single amino acid rAbcc6 mutants generated for our study are summarized and positioned in a topology model of rAbcc6 below (Figure 3A).

**Figure 4:**
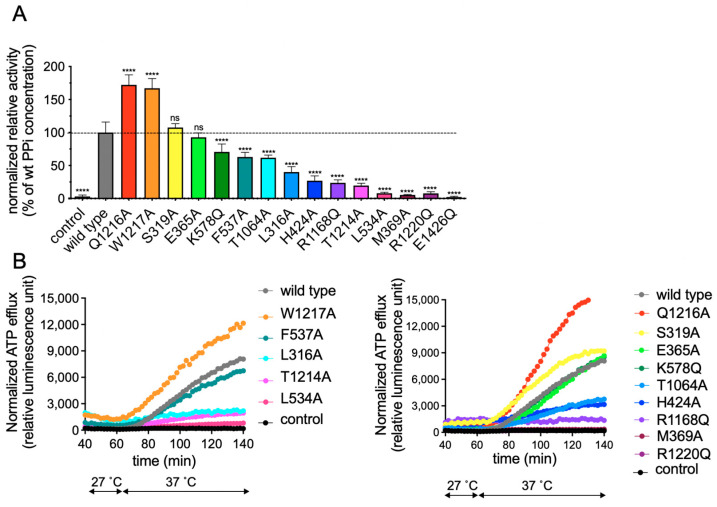
Activity of rAbcc6 mutants in HEK293 cells. (**A**): PPi accumulation in culture medium and (**B**): ATP efflux from cell lines overexpressing rAbcc6 in which amino acid residues corresponding to those forming the bAbcc1 LTC_4_ binding cavity were mutated. Data are presented as means ± SD for (**A**). For (**B**), means of representative experiments, each with at least 4 replicates are shown. In (**B**) data are presented in two graphs to better see results of individual mutants. Wild type: wild-type rAbcc6, control: parental HEK293 cells. The dashed line in (**A**) indicates the average amount of PPi in medium of HEK293 cells overproducing wild-type rAbcc6, which was set at 100%. Values have been adjusted to take any differences in protein expression of the mutants relative to wild type rAbcc6 into account. The same color coding was used for each mutant in panels A and B. **** *p* < 0.001 (ANOVA and subsequent Dunnett’s multiple comparison test). Changes were considered biologically relevant when reduced by >50% compared to wild-type rAbcc6.

Levels of the mutant rAbcc6 proteins in HEK293 cells varied but were within the same range as those of wild-type Abcc6 (Figure 3B). We then characterized the functionality of the rAbcc6 mutants by following PPi accumulation in the culture medium as an indirect measure of NTP release (Figure 4A,B), as well as by directly determining ATP efflux using a luciferin/luciferase-based assay (Figure 4B). In both assays the untransfected, parental, HEK293 cell line as well as the cell line expressing the catalytically inactive E1426Q mutant, did not release substantial amounts of ATP into the culture medium. In contrast, cells overproducing wild-type rAbcc6 released large amounts of ATP, resulting in robust PPi accumulation in the culture medium (Figure 4). These results demonstrate the suitability of these assays for measuring the consequences of the mutations introduced into rAbcc6.

**Figure 5:**
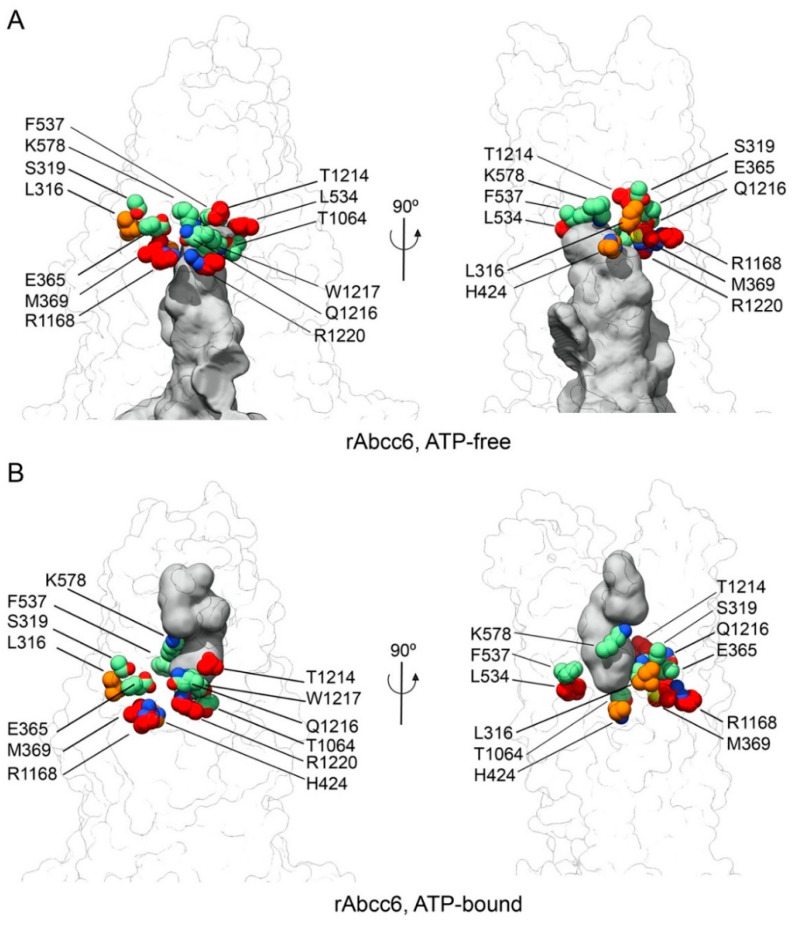
Mutation of the amino acids forming the modelled rAbcc6 substrate-binding cavity affect ATP efflux to different degrees. View of the (**A**) ATP-free inward-facing and (**B**) ATP-bound, outward-facing models of rAbcc6. rAbcc6 residues that when mutated abolished and reduced ATP efflux are shown as red and orange spheres, respectively. Residues that once mutated did not affect ATP efflux are shown as green spheres. The protein is shown as a white transparent surface and the volume of the main cavity in both models is shown as a gray surface. The volume was calculated with the 3V webserver [34].

Many of the rAbcc6 single amino acid mutants allowed cellular ATP efflux similar to that seen for wild-type rAbcc6, as determined by both PPi accumulation in the medium (Figure 4A) and the direct ATP efflux assay (Figure 4B). ATP release was substantially reduced (>75%) when M369, L534, R1168, T1214, and R1220 were mutated (Figure 4A,B). Changing L316 and H424 residues into alanine moderately reduced (>50%) efflux activity of rAbcc6. The substitution of the other residues did not reduce, or less substantially reduced, ATP efflux. The two arginine residues critical for function (rAbcc6 R1168 and R1220) belong to TMD2. In the ATP-free, LTC_4_-bound, inward-facing conformation, R1220 (TM17) localizes near one of the entrances of the modelled substrate-binding cavity, lined by TM15 and TM17 (Figure 5A). M369 (TM7), L534 (TM10), and R1168 (TM16) approximately lie on the same plane as R1220, and their side chains are exposed to the main cavity, with L534 located on the opposite side (Figure 5A). Among the residues that abolish ATP efflux when mutated, T1214 (TM17) is the one located further up in the transmembrane cavity (Figure 5A). In this conformation, among the other residues considered in this study, the side chains of L316 (TM6) and S319 (TM6) are those further away from the main cavity (Figure 5A). In the outward-facing, ATP-bound state, the cavity opens towards the opposite side of the membrane. In this state many of the residues are more buried and located towards the bottom of the outward-facing cavity (Figure 5B). As mentioned earlier, R1168 and R1220 are conserved among all the sequences considered for model building (see Supporting Information files TMD1-NBD1_Alignment.pdf and TMD2-NBD2_Alignment.pdf), as well as all ABCC family members and their orthologs. The analogous amino acids are indispensable for all the transport activities of hABCC1, suggesting a key function in overall protein structure [32,33], although we cannot completely rule out interaction with its substrates. M369 is present as a phenylalanine and a tryptophan in the ABCC1 and ABCC5 sequences, respectively, while rAbcc6 L534 is a threonine in ABCC1 and a valine in ABCC5 (see Supporting Information file TMD2-NBD1_Alignment.pdf). rAbcc6 T1214 is also not conserved among the sequences here considered, and it is present primarily as a tyrosine in ABCC6 and as a leucine in ABCC5 sequences (see Supporting Information file TMD2-NBD2_Alignment.pdf).

### 2.3. Subcellular Localization of the Single Amino Acid rAbcc6 Mutants That Showed Reduced ATP Efflux Activity

**Figure 6:**
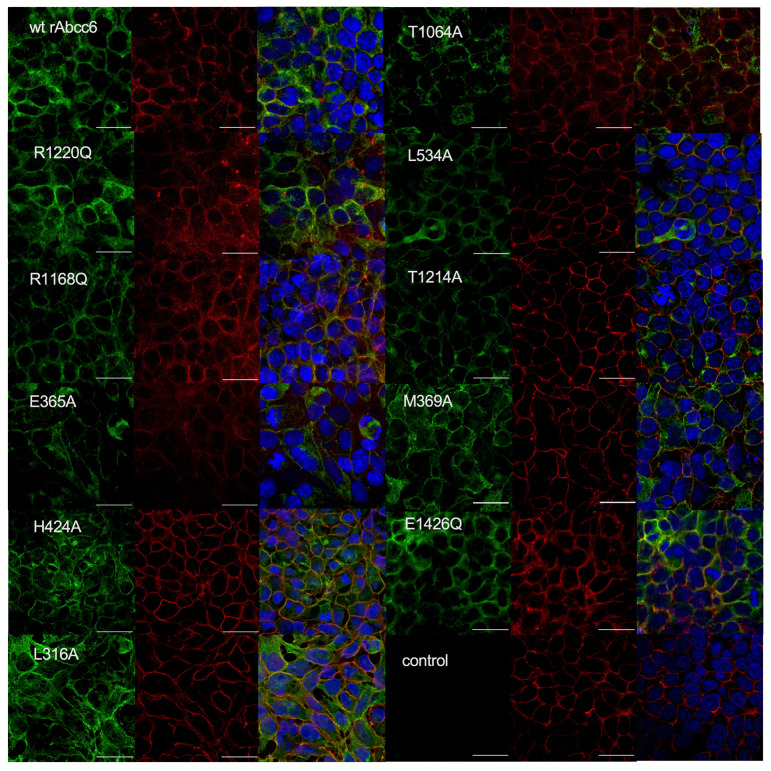
Subcellular localization of rAbcc6 mutants with reduced ATP efflux activity. Representative images of the subcellular localization of wild-type and single mutant rAbcc6 in HEK293 cells, as determined by confocal microscopy using the K14 anti-rAbcc6 rabbit polyclonal antibody. Red: Na^+^/K^+^ -ATPase, a marker for the plasma membrane; Green: rAbcc6.; Blue: DAPI nuclear staining; wt rAbcc6: wild-type rAbcc6, control: parental HEK293 cells. All scale bars represent 30 µm.

To exert its function, ABCC6 needs to reside in the plasma membrane. Confocal microscopy demonstrated that all rAbcc6 mutants with reduced activity routed to the plasma membrane, similar to wild-type rAbcc6 (Figure 6). This indicates that reduced plasma membrane localization was not the underlying cause of the reduced activity of these rAbcc6 mutants. Notably, although a significant proportion of the rAbcc6 mutant proteins also resided in intracellular compartments, this was not different from wild-type rAbcc6 and is consistent with our previous observations [19].

### 2.4. Functional Consequences of Changing All 11 Amino Acids of the Modeled rAbcc6 Substrate-Binding Site into Those That Comprise the bAbcc1 LTC_4_ Binding Site

As most mutants with single amino acid changes in the modeled rAbcc6 substrate binding cavity retained at least 25% activity, we wondered what the consequences would be if the entire modeled substrate binding cavity was altered such that it more closely mimicked that of ABCC1. Of the fourteen amino acids corresponding to those in the bAbcc1 cryoEM structure forming the LTC_4_-binding cavity, three are identical in rAbcc6 (rAbcc6 R1168, W1217 and R1220) (Table 1 and Figure 2). Thus, the remaining eleven amino acids of the modeled substrate-binding cavity were changed into residues found in bAbcc1 at the same positions (L316K, S319H, E365L, M369F, H424Y, F537W, L534T, K578F, T1064M, T1214Y, and Q1216N) to generate a rAbcc6 mutant protein we have termed rAbcc6-11aa.

**Figure 7:**
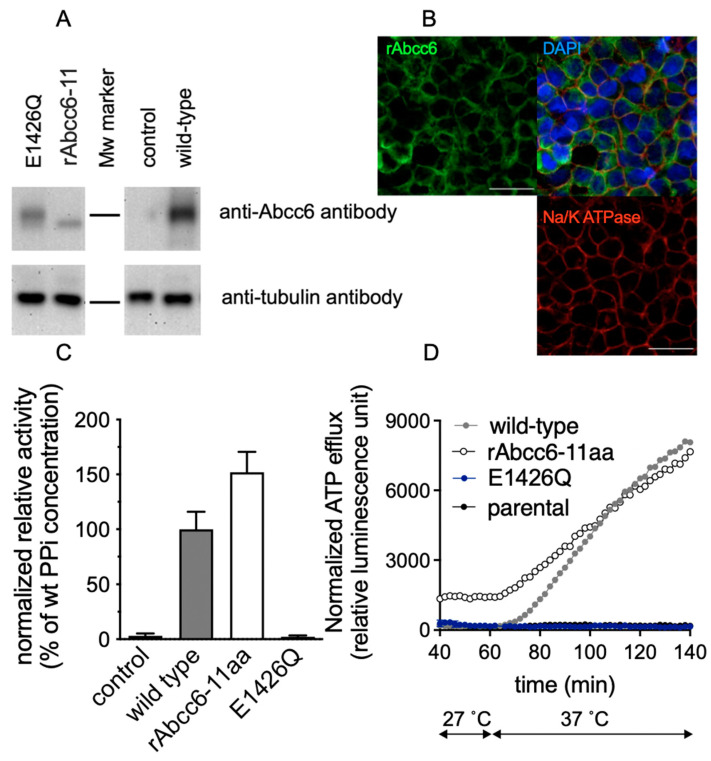
Expression, activity, and subcellular localization of the rAbcc6-11aa mutant. (**A**): Detection of rAbcc6-11aa in HEK293 cells by immunoblot analysis. (**B**): Subcellular localization of rAbcc6-11aa in HEK293 cells. Red: Na^+^/K^+^ -ATPase, a marker for the plasma membrane; Green: rAbcc6; Blue: DAPI. All scale bars represent 30 µm. (**C**): PPi accumulation in the medium of the indicated HEK293 cell lines (**D**): ATP efflux from the indicated HEK293 cell lines. Data are presented as means ± SD for (**C**), while means of representative experiments with at least 4 replicates are shown for (**D**). wild-type: wild-type rAbcc6, control: parental HEK293 cells. The dashed line in (**C**) indicates the average PPi level in medium of HEK293 cells overproducing wild-type rAbcc6, which was set at 100%. The slight differences in electrophoretic mobility of some of the mutants may be attributed to altered glycosylation or other post-translational modification. In panels C and D values have been adjusted to take differences in protein expression of the mutants relative to wild type rAbcc6 into account.

As shown in Figure 7A, the rAbcc6-11aa was expressed at about 6.5-fold lower levels than the wild-type rAbcc6 protein when overexpressed in HEK293 cells and appeared to have a faster electrophoretic mobility (Figure 7A). The reason for the altered mobility of rAbcc6-11a is not known but may be due to changes in glycosylation or other post-translational modification. If underglycosylated, the mutant rAbcc6-11aa protein may have a faster turn-over time thus explaining why it is expressed at lower levels than the wild-type protein. However, even if the mutations caused some misfolding of the transporter during its biosynthesis that impaired its glycosylation, the altered protein structure has remained stable enough to traffic to the plasma membrane where it can still carry out active transport. Thus, rAbcc6-11aa still mediated ATP release, as illustrated by both PPi accumulation in the medium and by real-time ATP efflux assays (Figure 7C,D, respectively). After adjustment for lower protein levels, rAbcc6-11aa seemed to even display higher activity than the wild-type protein. These data indicate that changing these 11 amino acids may have affected the stability of rAbcc6 but had minimal effect on the intrinsic activity of the protein. Consistent with the significant activity of the rAbcc6-11aa mutant protein, its abundance in the plasma membrane was also relatively high (Figure 7B).

We next set out to test if the rAbcc6-11aa mutant with the hABCC1/bAbcc1 LTC_4_-binding cavity residues transports LTC_4_. Of note, an initial characterization of hABCC6 indicated it could transport LTC_4_ [6], although this was not the case for its ortholog rAbcc6 [35]. Nevertheless, we reasoned that introducing the amino acids in bAbcc1 thought to form the bipartite LTC_4_-binding cavity at the corresponding positions in rAbcc6 might establish LTC_4_ transport in the latter ABC transporter. The cellular ATP efflux capacity of the rAbcc6-11aa indicated the protein retained activity. However, in vitro vesicular transport experiments (widely held to be the gold-standard to confirm that a molecule is a substrate of a specific ABC transporter) failed to demonstrate LTC_4_ transport by the rAbcc6-11aa protein (data not shown). Low levels of LTC_4_ transport might have been missed, however, because of the low expression levels of rAbcc6-11aa in our system (HEK293 cells).

## 3. Discussion

ABC transporters use the energy derived from ATP hydrolysis to mediate transport of a wide range of substrates across membranes. Several members of the ABCC subfamily have been studied for their role in drug resistance and human diseases. Many of these transporters translocate negatively charged solutes. For example, ABCC1 transports organic anions, including LTC_4_, a cysteinyl leukotriene with proinflammatory properties [26,27,36], while ABCC5 transports cyclic nucleotides such as cAMP and cGMP [37,38], important for signal transduction. Most of the available data indicate ABCC6 transports ATP and other NTPs out of cells [10,11,39,40]. However, even now, ABCC6-mediated ATP efflux has not been shown in vesicular uptake experiments, widely used to demonstrate that a given compound is actively transported by an ABC transporter [6,19]. Being involved in the efflux of ATP confers a unique physiological role for ABCC6 among ABCC transporters [19]. We hypothesized that the substrate-binding cavity of ABCC6 is also unique among ABC transporters, involving amino acids that are not necessarily evolutionarily conserved among members of the ABCC subfamily. To address this, we built homology models of hABCC6/rAbcc6 in two different conformations (ATP-free, inward-facing and ATP-bound, outward-facing) (Figure S1). The previous homology modeling studies of ABCC6 were performed using available structures of bacterial ABC transporters or the related mouse P-glycoprotein, a member of the ABCB subfamily [41,42]. Here, we used the cryoEM structures of bAbcc1 [22,23], and compared the residues in the bipartite LTC_4_ binding pocket of bAbcc1 with other ABCC1 sequences, as well as ABCC6 and ABCC5 sequences (see the Supporting Information alignment files). This choice was dictated by the shared ability of ABCC1, ABCC5 and ABCC6 transporters to translocate negatively charged substrates including cyclic nucleotides, as well as an early study reporting the ability of hABCC6 to transport LTC_4_ [20], which suggested there may be similarities in substrate recognition by hABCC6, hABCC1, and ABCC5. Of note, however, is that rAbcc6 has never been shown to transport LTC_4_ [35] and later studies also failed to confirm LTC_4_ transport by hABCC6 [6]. Nevertheless, the overall electrostatic properties of the transmembrane cavity appear remarkably similar between the hABCC6/rAbcc6 models and the bAbcc1 cryoEM structures, with a strong positive potential that might contribute to the driving force for the uptake of negatively charged substrates from the cytosol (Figure 1 and Figure S2). Interestingly, the negative potential on the extracellular end of the TMDs (Figure S2A) appeared less prominent following the conformational change to the ATP-bound state (Figure S2B), possibly facilitating the negatively charged ATP to leave the substrate binding cavity. Among the bAbcc1 residues found in the proposed LTC_4_-binding cavity (Table 1) are three residues that are identical to the corresponding R1168, W1217, and R1220 in rAbcc6 (Figure 2 and Figure S4), which are conserved among all human ABCC transporters (Figure S4) and the sequences considered in this study for model building, likely indicating a common function in maintaining the integrity of the substrate binding cavity. In the ATP-free, inward-facing homology model, their side chain faces the main cavity, and the residues belong to TM helices 16 and 17 of TMD2, known to be crucial for substrate binding and transport in ABCC1 [32,43]. Interestingly, in ABCC1 substitutions of W1246 (corresponding to rAbcc6 W1217) adversely affects transport of estradiol-17β-glucuronide and methotrexate but not of LTC_4_ [29,43], also implicating a role of this amino acid in transporter substrate selectivity. Furthermore, even conservative same charge substitutions of R1197 and R1249 in hABCC1 cause a global loss of transport activity [32].

We proceeded with functional studies to test if the putative transmembrane ATP-binding site of ABCC6 overlaps with the LTC_4_-binding cavity of bAbcc1 using a rAbcc6 model system [22]. Of the fourteen single amino acid changes introduced into the putative ABCC6 substrate binding cavity, five were found to reduce ABCC6-dependent ATP release by >75%, M369, L534, R1168, T1214, and R1220. Mutating the amino acid residue corresponding to rAbcc6 L534 in ABCC1 (T550) did not affect organic anion transport [44]. In contrast, even conservative mutations of hABCC1 F385 and Y1243 [45] (corresponding to M369 and T1214 in rAbcc6) adversely affected the transport capacity of one or more organic anions by hABCC1 (Unpublished, Conseil and Cole). Regarding the rAbcc6 R1168 position, previous studies (Table S2) have shown that opposite charge but also like-charge substitutions of hABCC1 at R1197, corresponding to rABCC6 R1168 and hABCC6 R1169, respectively, substantially reduced overall organic anion transport activity (all 4 organic anion substrates tested) as well as LTC_4_ binding [32]. Regarding rAbcc6 R1220, mutations of the corresponding R1221 in hABCC6 have been reported to be disease causing (R1221C) [46,47,48] and pathogenic (R1221H) [47,49]. The corresponding amino acid in hABCC1, R1249, is crucial for overall organic anion transport activity as well, not just glutathione-dependent binding of substrates and transport of LTC_4_ [32,33]. In hABCC2, the analogous amino acid R1257 is also indispensable for activity, as the mutant protein is deficient in glutathione conjugate transport, despite correctly routing to the plasma membrane [50]. In hABCC4, substitution of R998, which is analogous to R1221 in hABCC6 (and R1249 in hABCC1), by alanine completely abolishes the transport of cyclic guanosine monophosphate (cGMP) [51]. Based on the fact that the rAbcc6 R1168 and R1220 are highly conserved in ABCC1-6 and that the mutation of these residues hampers the transport function in the paralogs, we consider it likely that the presence of the charged residues at these positions is indispensable for all ABCC proteins and the requirement for a positive charged residue at this position is not specific to ABCC6. The other residues corresponding to those that form the binding cavity for LTC_4_ in bAbcc1 had less impact on the rAbcc6-mediated ATP efflux. Of these, only L316A and H424A had activity that was <50% that of wild-type rAbcc6 (Figure 4). Somewhat surprisingly, despite evidence of misfolding, the mutant rAbcc6-11aa protein containing the same LTC_4_ binding cavity amino acids as bAbcc1 was still functional and able to efflux ATP. This suggests that the amino acids corresponding to those proposed to form the bAbcc1 LTC_4_ binding cavity, in ABCC6/rAbcc6 are not essential for interaction with or the recognition of its physiological substrate, ATP. Our conclusion, therefore, is that the binding site for ATP in the transmembrane cavity of ABCC6 is clearly distinct from the LTC_4_ binding cavity in bAbcc1. The possible exceptions are two highly conserved positively charged residues described above, namely rAbcc6 R1168 and R1220, which are common to the substrate-binding cavity of the ABCC transporters characterized and likely are essential for proper folding and assembly into a transport competent protein. Despite the fact that ABCC1 and ABCC6 arose from a recent gene duplication, simple evolution of a common substrate-binding site most likely does not explain the structurally very distinct substrates effluxed by the two proteins.

The molecular details of ABCC6-mediated cellular ATP release remain unknown. As outlined above, an attractive hypothesis is that ABCC6 functions as an ATP-dependent ATP efflux pump. There are three sets of observations that support the idea that ABCC6 is an ATP efflux pump. First, most members of the C-branch of the ABC superfamily, including ABCC6′s closest homolog ABCC1, are bona fide organic anion efflux transporters [24,26,52] and ABCC6 has been shown to transport several organic anions in vitro, albeit sluggishly [6]. Second, the ATP efflux rates found in HEK293-ABCC6 cells [10] are compatible with a direct transport mechanism for ATP as these rates are very similar to the rates by which ABCC1, ABCC2, and ABCC3 pump morphine-3-glucuronide out of transfected HEK293 cells [18]. Third, ATP efflux from ABCC6-containing cells can be blocked by the general ABCC transport inhibitors benzbromarone, indomethacin and MK571 (data not shown).

As mentioned, vesicular transport experiments are often used to establish substrates of ABC transporters [24]. However, such assays have so far failed to directly demonstrate the ABCC6-dependent transport of radiolabeled ATP into inside-out membrane vesicles. We can, therefore, not completely exclude the possibility that ABCC6 mediates cellular ATP release other than by direct transport. Purification of ABCC6 and subsequent reconstitution in proteoliposomes should provide a cleaner experimental system to study ATP transport, for instance by using dual color fluorescence burst analysis (DCFBA) [53], a technique that has a more favorable signal-to-noise ratio than the standard vesicular transport assays that employ radiolabeled ATP. The elucidation of the molecular structure of ABCC6, for instance by cryoEM, might also in the future provide clues about the molecular mechanism by which ABCC6 mediates ATP release. Despite many years of intense work on ABCC6, this ABC protein has not given many of its secrets away.

## 4. Materials and Methods

### 4.1. Model Building

We built homology models for the structural ABC transporter core of hABCC6 and rAbcc6, including residues of TMD1, NBD1, TMD2, and NBD2. First, we generated a multiple sequence alignment (MSA) using MAFFT [54] including the sequences of the hABCC6, hABCC1, and hABCC5 proteins from multiple organisms (Table S1), retrieved from UniProtKB [55]. Based on the alignment of hABCC6 and rAbcc6 sequences with bAbcc1, homology models of hABCC6 and rAbcc6 were generated with Modeller 9v15 [56], using the inward- and outward-facing cryoEM structures of bAbcc1 as templates [22,23]. For hABCC6 and rAbcc6, 20 models were generated for both the inward- and the outward-facing states, by applying a slow refinement protocol and 20 cycles of simulated annealing as in our previous work on hABCC7 (CFTR) [57]. For the inward-facing state, the ABCC6 models were generated considering the presence of LTC_4_ in the template. The final hABCC6 and rAbcc6 models for each conformation were chosen based on the Discrete Optimized Protein Energy (DOPE) value implemented in Modeller. The rAbcc6 and hABCC6 models are provided in the Supporting Information as PDB files. The MSA is provided in the form of two separate files, covering residues of TMD1 and NBD1 (see TMD1-NBD1_Alignment.pdf), and residues of TMD2 and NBD2 (see TMD2-NBD2_Alignment.pdf). These files were generated using Jalview [58] and the residues are colored according to the Clustal X coloring scheme implemented in Jalview. The residues highlighted in bold correspond to the residues investigated in the present study and the rAbcc6 numbering of amino acids is indicated. The residues of the TM helices of TMD1 and TMD2 are also annotated. The percentage amino acid identity among bAbcc1, rAbcc6, and hABCC6 was calculated on the TMD1-NBD1 and TMD2-NBD2 alignment using the id_table command available in Modeler. Sequences of the hABCC1 and hABCC6 proteins shown in Figure S5 were retrieved from UniProtKB [55] and were aligned using Clustal Omega [59].

Electrostatic potential calculations were performed using the PDB2PQR and APBS webservers [60,61], using a pH of 7 and a NaCl concentration of 0.15 M. The electrostatic potential was visually mapped on the molecular surface of the models using UCSF Chimera [62], with a surface offset parameter of 1.4. Figure 5 was also generated using UCSF Chimera, after calculating the cavity volumes with the 3V webserver, using the default parameters for the Channel Finder module [34]. Other figures were generated using PyMOL [63].

### 4.2. Mutagenesis

Mutagenesis was performed as described previously [19]. Briefly, mutations were introduced into the Gateway entry vector pEntr223-rAbcc6 by uracil-specific excision reagent (USER) cloning with the primers listed in Table 2 using Phusion U PCR master mix (Thermo Scientific, Waltham, MA, USA). PCR fragments were purified using the Nucleospin gel and PCR cleanup kit (Macherey-Nagel, Düren, Germany) and assembled using the USER enzyme mix (New England Biolabs, Ipswich, MA, USA), according to the manufacturer’s instructions. Resulting circular constructs were verified by Sanger sequencing and transformed into competent E. coli DH5alpha cells. The cDNAs encoding pEnter223-rAbcc6 mutants were subsequently subcloned into a Gateway compatible pQCXIP expression vector using LR Clonase-II (Thermo Scientific, Waltham, MA, USA).

### 4.3. Cell Culture and Generation of Mutant Cell Lines

Cell culture and generation of mutant cell lines were performed as described previously [14,19]. Briefly, HEK293 cells overproducing wild-type rAbcc6 (HEK293-rAbcc6) and control, untransfected cells (HEK293-control) were cultured at 37 °C in a 5% CO_2_ atmosphere under humidifying conditions in DMEM (HyClone, GE Healthcare, Chicago, IL, USA) with 100 U pen/strep per mL (Gibco, Waltham, MA, USA) supplemented with 5% FBS (Fisher Scientific, Waltham, MA, USA). rAbcc6 mutants cloned into the pQCXIP expression vector were transfected into HEK293 cells using the calcium phosphate precipitation method. Transfected cells were selected in medium containing 2 µM puromycin. Cell lines were established from clones showing high expression of the respective rAbcc6 mutants. Of note, several clones were generated for each rAbcc6 mutation and these subclones behaved very similarly with respect to PPi accumulation in the culture medium.

### 4.4. Immunoblot Analysis of Wild-Type and Mutant rAbcc6

The expression of rAbcc6 was confirmed by immunoblot analysis as described previously [19]. Briefly, cell lysates were prepared in lysis buffer (0.1% Triton-x-100, 10 mM KCl, 10 mM Tris-HCl and 1.5 mM MgCl2, pH 7.4) supplemented with protease inhibitors (EDTA-free Protease Inhibitor Cocktail, Sigma Aldrich, St. Louis, MO, USA. Samples containing 5 µg of total protein determined by BCA assay were separated on 7.5% SDS-polyacrylamide gels (Bio-Rad, Hercules, CA, USA and transferred to a PVDF membranes using a semi-dry blotting system (Trans-Blot Turbo, Bio-Rad, Hercules, Ca, USA). rAbcc6 was detected with the polyclonal K14 rabbit anti-rAbcc6 antibody (diluted 1:3000) (kind gift of Dr. Bruno Stieger) and horseradish peroxidase (HRP)-conjugated donkey anti-rabbit secondary antibody (1:5000) (Fisher Scientific, Waltham, MA, USA). Mouse anti-α-tubulin (1:1000) (Sc-23948, Santa-Cruz Biotechnology, Dallas, TX, USA) followed by HRP-conjugated polyclonal rabbit anti-mouse IgG employed as secondary antibody (1:5000) (P0161, Dako, Agilent, Santa Clara, CA, USA), was used as a protein loading control. Antibody binding was visualized by ECL (Pierce Western blotting substrate, Thermo Scientific, Waltham, MA, USA).

### 4.5. Subcellular Localization of rAbcc6 in HEK293 Cells

The localization of rAbcc6 in intact HEK293 cells was detected as described previously [19]. Briefly, HEK293 cells were seeded and grown for 2 days on 4 well μ-Slides (ibiTreat 1.5 polymer coverslip, 80426, Ibidi) coated with poly-D-lysine. The cells were fixed in 4% PFA and subsequently in −20 °C cold methanol for 5 min each and then samples were blocked with Protein Block (Fisher Scientific) for 60 min. Samples were then incubated for 60 min with the polyclonal rabbit anti-rAbcc6 antibody K14 diluted 1:100 and the mouse monoclonal anti-Na^+^/K^+^-ATPase antibody (ab7671, Abcam, Cambridge, UK) diluted 1:250. Then samples were incubated for 60 min with Alexa Fluor 488-conjugated goat anti-rabbit secondary antibody (A11008, Fisher Scientific, Waltham, MA, USA) and Alexa Fluor 568 conjugated goat anti-mouse antibody (A11004, Fisher Scientific, Waltham, MA, USA), both diluted 1:1000. The samples were subsequently incubated with 300 nM DAPI for 5 min to stain nuclei. The subcellular localization of wild-type and mutant forms of rAbcc6 was then analyzed using a Nikon (Tokyo, Japan) Eclipse Ti two point-scanning laser confocal microscope equipped with a Nikon A1R+. A Plan Fluor 40× Oil DIC H N2 objective with a 1× optical zoom was used with 405.5, 490.0 and 561.3 nm excitation and 450/50, 525/50 and 595/50 nm emission filters, respectively. Images were acquired with the pinhole set to 1 airy unit.

### 4.6. Quantification of PPi Levels in Medium Samples

We have previously found that HEK293 cells endogenously express ENPP1 [10] and that PPi accumulation in the medium can be used as a robust secondary assay to determine the amount of ATP released by cells into the culture medium. Using two independent assays to follow ATP efflux provides robust data on the activity of the studied rAbcc6 mutants. PPi concentrations in cell culture medium samples were determined as described previously [14,19]. Briefly, PPi was quantitatively converted into ATP by incubating samples and standards in an assay containing 50 mM HEPES pH 7.4, 80 µM MgCl2, 32 mU/mL ATP sulfurylase (New England Biolabs, Ipswich, MA, USA), and 8 µM adenosine 5’-phosphosulfate (Santa Cruz Biotechnology, Dallas, TX, USA) at 30 °C for 30 min and the reaction was terminated by incubating the samples at 90 °C for 10 min. ATP levels were then determined in the reaction mix by bioluminescence by adding BacTiterGlo reagent (Promega, Madison, WI, USA) in a 1:1 ratio in a total volume of 40 µL. PPi concentrations in medium samples were then calculated by interpolation from a standard curve. The values were adjusted by subtracting background provided by controls in which ATP sulfurylase was omitted.

### 4.7. Real-Time ATP Efflux Assay

The ability of the transfected HEK293 cells to release ATP into the culture medium was determined using confluent monolayers as described previously [14,19]. In brief, the medium was removed and replaced with 50 μL efflux buffer, consisting of 11.5 mM HEPES (pH 7.4), 130 mM NaCl, 5 mM MgCl2, 1.5 mM CaCl2, and 11.5 mM glucose. The cells were then incubated for 1 hr at 27 °C. Next, 50 µL BactiterGlo reagent (Promega) dissolved in efflux buffer was added to each well. Bioluminescence was subsequently determined in real time in a Flex Station 3 microplate reader (Molecular Devices, San Jose, CA, USA) as detailed previously [14,19]. The real-time ATP efflux assay was run at 27 °C for the first 1 h and then at 37 °C for 2 hr. The initial low temperature allowed endogenous ecto-nucleotidases to degrade the Abcc6-independent background ATP efflux induced by the medium change.

## Figures and Tables

**Table 1:** Amino acid residues in rat and human ABCC6, human ABCC1 and human ABCC5 at the same positions proposed to form the LTC_4_ binding cavity in bAbcc1. In the last column it is indicated to which transmembrane helix (TM) and transmembrane domain (TMD) the residues belong. bAbcc1, bovine Abcc1; hABCC1, human ABCC1; hABCC6, human ABCC6; rAbcc6, rat Abcc6; hABCC5, human ABCC5.

bAbcc1	hABCC1	hABCC6	rAbcc6	hABCC5	TM Helix
K332	K332	L318	L316	L186	TMD1, TM6
H335	H335	S321	S319	T189	TMD1, TM6
L381	L381	E367	E365	L236	TMD1, TM7
F385	F385	M371	M369	W240	TMD1, TM7
Y440	Y440	Y426	H424	V293	TMD1, TM8
T550	T550	L536	L534	V403	TMD1, TM10
W553	W553	F539	F537	A406	TMD1, TM10
F594	F594	K579	K578	Q432	TMD1, TM11
M1092	M1093	S1065	T1064	M992	TMD2, TM14
R1196	R1197	R1169	R1168	R1096	TMD2, TM16
Y1242	Y1243	T1215	T1214	L1142	TMD2, TM17
N1244	N1245	Q1217	Q1216	Q1144	TMD2, TM17
W1245	W1246	W1218	W1217	F1145	TMD2, TM17
R1248	R1249	R1221	R1220	R1148	TMD2, TM17

**Table 2:** Primers used to generate the various rAbcc6 mutants.

Construct	Mutation	Forward Primer	Reverse Primer
**rAbcc6-L316A**	L316A	AGCGCCGUCATTAGCGATGCCTTCAGGTTTG	ACGGCGCUGAGGGTCCCCAGCAGGAAA
**rAbcc6-S319A**	S319A	ATTGCCGAUGCCTTCAGGTTTGCTGTT	ATCGGCAAUGACCAGGCTGAGGGTCCC
**rAbcc6-E365A**	E365A	ACTGTTUGCCCAGCAGTACATGTACAGA	AAACAGUGTCTGTAGGCAGGCCGACAA
**rAbcc6-M369A**	M369A	AGTACGCCUACAGAGTCAAGGTCCTGCAGATG	AGGCGTACUGCTGTTCAAACAGTGTCTG
**rAbcc6-H424A**	H424A	ATCCTCGCCCUCAACGGGCTGTGGCTGCTCTT	AGGGCGAGGAUGCTCTCGACCAGCCGCTG
**rAbcc6-L534A**	L534A	AAGTGTCUACATTTCTGGTGGCGCTGGTTGT	AGACACTUGGAAGGACACGGCAGACACAGAGAAGAGGAAG
**rAbcc6-F537A**	F537A	AAGTGTCUACATTTCTGGTGGCGCTGGTTGT	AGACACTUGGGCGGACACGAGAGACACAGA
**rAbcc6-K578Q**	K578Q	AGCCAGGCCUTCCTCCCCTTCTCTGTGC	AGGCCTGGGCUTGGTTAAGGATG
**rAbcc6-T1064A**	T1064A	AGGGCCCUGCTGACCTATGCCTTTGG	AGGGCCCUCATCTTGTCTGGGATGTCCACAT
**rAbcc6-R1168Q**	R1168Q	ACCAGTGGCUGGCTGCCAACCTGGAGCT	AGCCACTGGUCAGCCACCAGCCTCGGGA
**rAbcc6-T1214A**	T1214A	AGGCTCUGCAGTGGGTGGTCCGCAGCTG	AGAGCCUGTGTTACCTGGAGGGCAGCAGAAACCG
**rAbcc6-Q1216A**	Q1216A	ACTCTGGCCUGGGTGGTCCGCAGCTGGAC	AGGCCAGAGUCTGTGTTACCTGGAGGGC
**rAbcc6-W1217A**	W1217A	AGGCCGUGGTCCGCAGCTGGACAGATC	ACGGCCUGCAGAGTCTGTGTTACCT
**rAbcc6-R1220Q**	R1220Q	AGTGGGTGGUCCAATCTGGAGAACAG	ACCACCCACUGCAGTCTGTGTTACCT
**rAbcc6-11AA**	L316K & S319H	AGGTCATUCACGATGCCTTCAGGTTTGCTGTTCCCAAGC	AATGACCUTGCTGAGGGTCCCCAGCAGGAAAGT
	E365L & M369F	AGCAGTACUTCTACAGAGTCAAGGTCCTGCAGATGAGGCTG	AGTACTGCUGCAGAAACAGTGTCTGTAGGCAGGCCGACAAG
	H424Y	ATCCTCUACCTCAACGGGCTGTGGCTGC	AGAGGAUGCTCTCGACCAGCCGCTG
	L534T	ACCGTGUCCTGGCAAGTGTCTACATTTCTGGTGGC	ACACGGUAGACACAGAGAAGAGGAAGGCGGAGGTCT
	F537W	AAGTGTCUACATTTCTGGTGGCGCTGGTTG	AGACACTUGCCAGGACACGAGAGACACAGAGAAGAGGAAGGC
	K578F	ATCCTTAACUTCGCCCAGGCCTTCCTCCCCTTC	AGTTAAGGAUGCTGAGCACCGTGAGCGT
	T1064M	AGGATGCUGCTGACCTATGCCTTTGGACTCCTGG	AGCATCCUCATCTTGTCTGGGATGTCCACATCCAC
	T1214Y	AGTATCUGAACTGGGTGGTCCGCAGCTGG	AGATACUGTGTTACCTGGAGGGCAGCAGAAACCG
	Q1216N	AACTGGGUGGTCCGCAGCTGGACAGATC	ACCCAGTUCAGAGTCTGTGTTACCTGGAGGGCAGC

## Data Availability

Not applicable.

## Supplementary Materials

The following are available online at https://www.mdpi.com/article/10.3390/ijms22136910/s1, Figure S1: Structural models of rABCC6 and hABCC6. Figure S2: Electrostatic potential at the extracellular end of the TMDs for ABCC6 and ABCC1, Figure S3: The LTC_4_ binding cavity in bABCC1 and hABCC6, Figure S4: Alignment of ABCC1-6 amino acid sequences. Table S1: List of the ABCC1 and ABCC6 sequences and their corresponding UniProtKB codes used to generate the alignment for the homology models. Table S2: Functional consequences of mutating rAbcc6 R1168 and R1220 and corresponding residues in hABCC6, hABCC1 and hABCC2. Supporting information files: TMD1-NBD1_Alignment.pdf, TMD2-NBD2_Alignment.pdf, hABCC6_inward-facing.pdb, hABCC6_outward-facing.pdb, rABCC6_inward-facing.pdb, rABCC6_outward-facing.pdb.
