# Supplementary material for: Mutagenic Analysis of the Putative ABCC6 Substrate-Binding Cavity Using a New Homology Model
Source: Int J Mol Sci. 2021 Jun 27;22(13):6910. doi: 10.3390/ijms22136910 (PMC8267652; doi:10.3390/ijms22136910)
Supplement: Supplementary file 1 [file TMD2-NBD2_Alignment.pdf › TMD2-NBD2_Alignment.pdf]

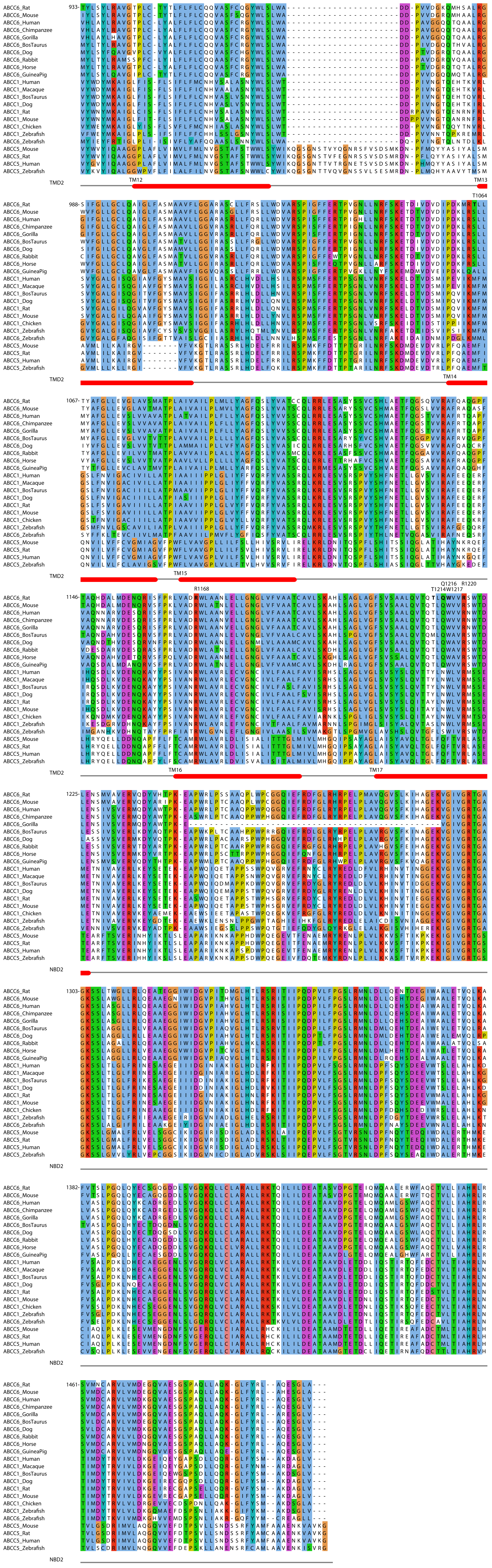
ABCC6_Rat
ABCC6_Mouse
ABCC6_Human
ABCC6_Chimpanzee
ABCC6_Gorilla
ABCC6_BosTaurus
ABCC6_Dog
ABCC6_Rabbit
ABCC6_Horse
ABCC6_GuineaPig
ABCC1_Human
ABCC1_Macaque
ABCC1_BosTaurus
ABCC1_Dog
ABCC1_Rat
ABCC1_Mouse
ABCC1_Chicken
ABCC1_Zebrafish
ABCC6_Zebrafish
ABCC5_Mouse
ABCC5_Rat
ABCC5_Human
ABCC5_Zebrafish
933
988
1067
1146
1225
1303
1382
1461
TMD2
TM12
TM13
T1064
TM14
TM15
R1168
Q1216
R1220
T1214W1217
TM16
TM17
NBD2
