# Supplementary material for: Mutagenic Analysis of the Putative ABCC6 Substrate-Binding Cavity Using a New Homology Model
Source: Int J Mol Sci. 2021 Jun 27;22(13):6910. doi: 10.3390/ijms22136910 (PMC8267652; doi:10.3390/ijms22136910)
Supplement: Supplementary file 1 [file ijms-22-06910-s001.zip › Supplemental information.pdf]

Table S1.
List of the ABCC1, ABCC5 and ABCC6 sequences and their corresponding UniprotKB codes used to generate the alignment for the homology models.

Name	UniProtKB code
ABCC6_Rat	O88269
ABCC6_Mouse	Q9R1S7
ABCC6_Human	O95255
ABCC6_Chimpanzee	A0A2I3SJS2
ABCC6_Gorilla	G3S3B7
ABCC6_Bovine	G5E62
ABCC6_Dog	F1PB44
ABCC6_Rabbit	G1SYV5
ABCC6_Horse	F7B7N5
ABCC6_Gpig	H0V465
ABCC1_Human	P33527
ABCC1_Macaque	Q864R9
ABCC1_BosTauros	Q8HXQ5
ABCC1_Dog	Q6UR05
ABCC1_Rat	Q8CG09
ABCC1_Mouse	O35379
ABCC1_Gallus	Q5F364
ABCC1_Zebrafish	A0A0R4IC81
ABCC6_Zebrafish	E7F118
ABCC5_Mouse	Q9R1X5
ABCC5_Rat	Q9QYM0
ABCC5_Human	O15440
ABCC5_Zebrafish	E1B2R7

TABLE S2. Functional consequences of mutating rAbcc6 R1168 and R1220 and corresponding residues in hABCC6, hABCC1 and hABCC2

ABCC6						hABCC1			hABCC2		
rAbcc6	mutation[a]	phenotype	hABCC6	variant[b]	ref	mutation	phenotype	ref	mutation	phenotype	ref
R1168	R1168A	ATP efflux reduced ≥75%[a]	R1169	-	-	R1197E<br>R1197K	Organic anion[c] transport reduced ≥80%	[1]	-	-	-
R1220	R1220A	ATP efflux reduced ≥90%[a]	R1221	R1221C	[2-4]	R1249A	LTC_4 transport reduced 80%; vincristine resistance abolished	[5]	R1257A	glutathione conjugate transport markedly (>50%) reduced	[6]
				R1221H	[4, 7, 8]	R1249K<br>R1249D	Organic anion[c] transport reduced ≥80%	[1]			

[a]this paper; [b]pathogenic hABCC6 variant associated with PXE; [c] organic anions tested included estradiol glucuronide, LTC_4, estrone sulphate, methotrexate

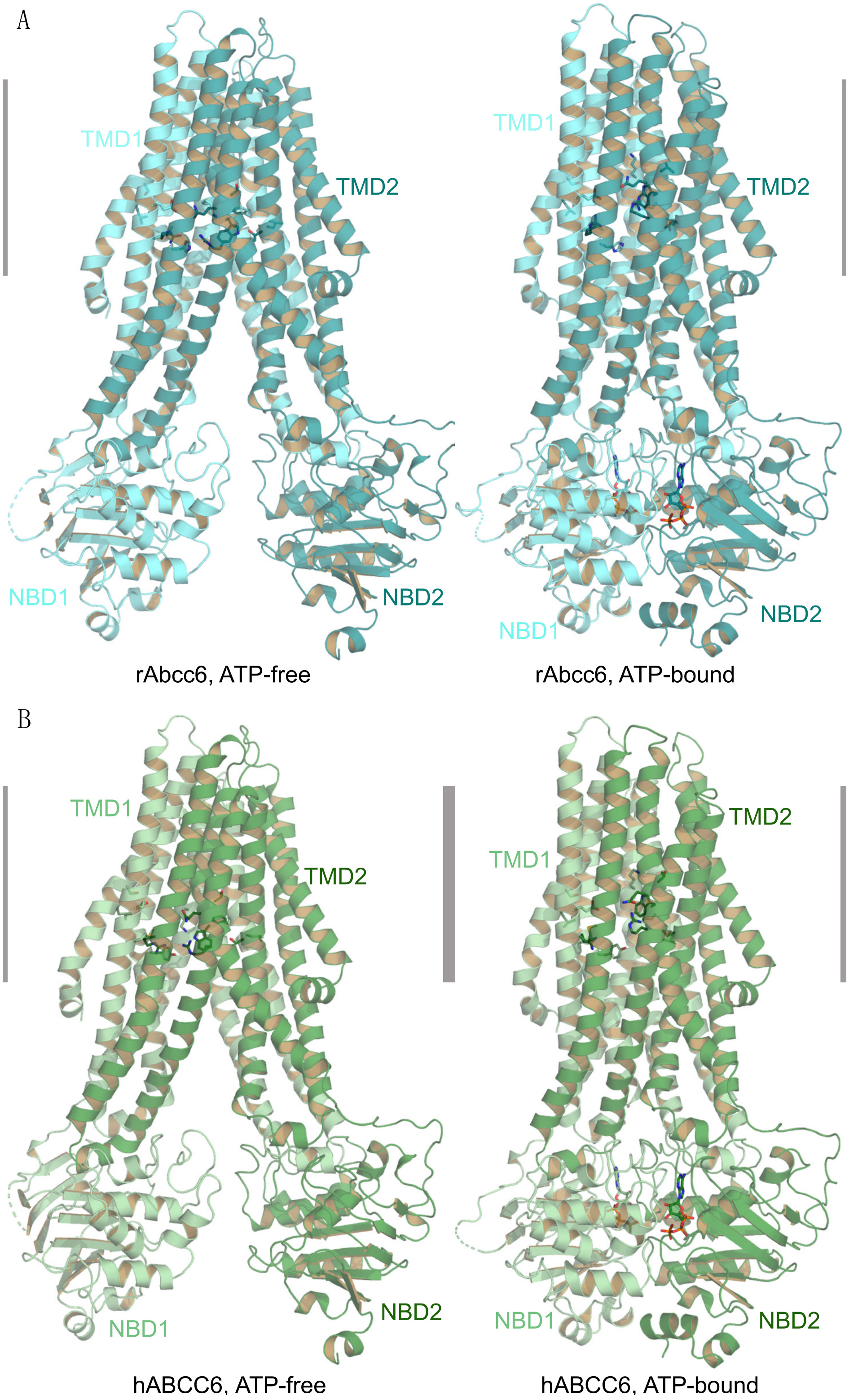

Figure S1. Structural models of rAbcc6 and hABCC6. The ABC core of ABCC6 (residues of TMD1-NBD1 and of TMD2-NBD2) is shown for (A) the rAbcc6 and (B) the hABCC6 ATP-free, inward-facing model (left panels) and ATP-bound, outward-facing conformational state of the transport cycle. The residues of the transmembrane cavity that are the subject of this study and described more in detail in the main text are shown as sticks. The ATP molecules bound to the NBDs in the outward-facing states are shown as sticks. TMD1, transmembrane domain 1; TMD2, transmembrane domain 2; NBD1, nucleotide binding domain 1; NBD2, nucleotide binding domain 2. The region of the TMDs embedded in the membrane is highlighted in gray.

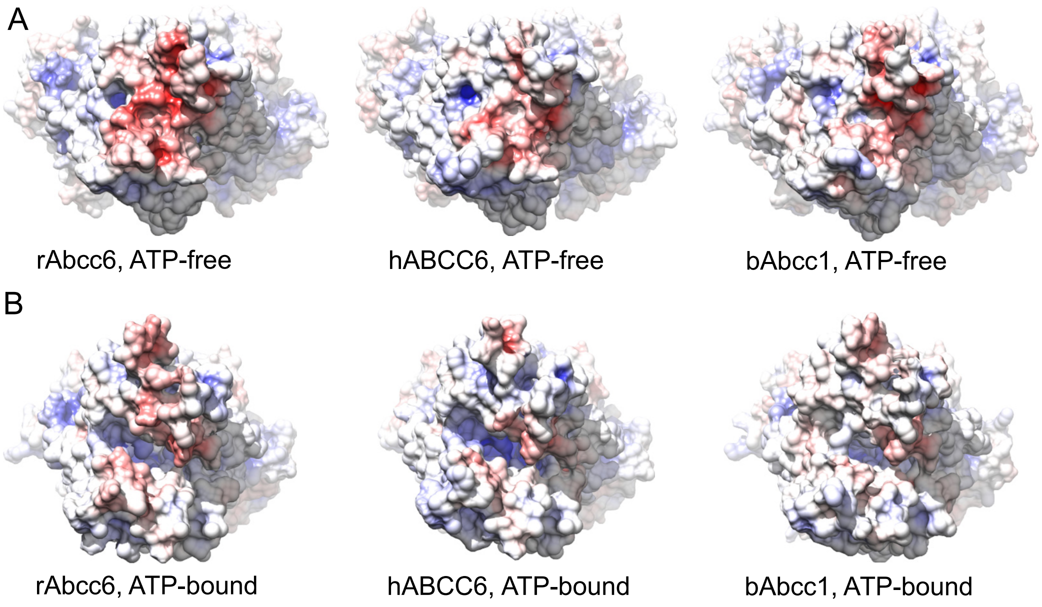

Figure S2. Electrostatic potential at the extracellular end of the TMDs for rAbcc6 hABCC6 and bAbcc1. View from the extracellular side of the electrostatic potential mapped on the molecular surface of (A) the ATP-free, inward-facing and (B) the ATP-bound, outward-facing homology models of rAbcc6 and hABCC6 and of the experimental structures of bAbcc1 [9, 10]. The isovalue was set at −10 k_BT/e for the negative potential (red) and +10 k_BT/e for the positive potential (blue).

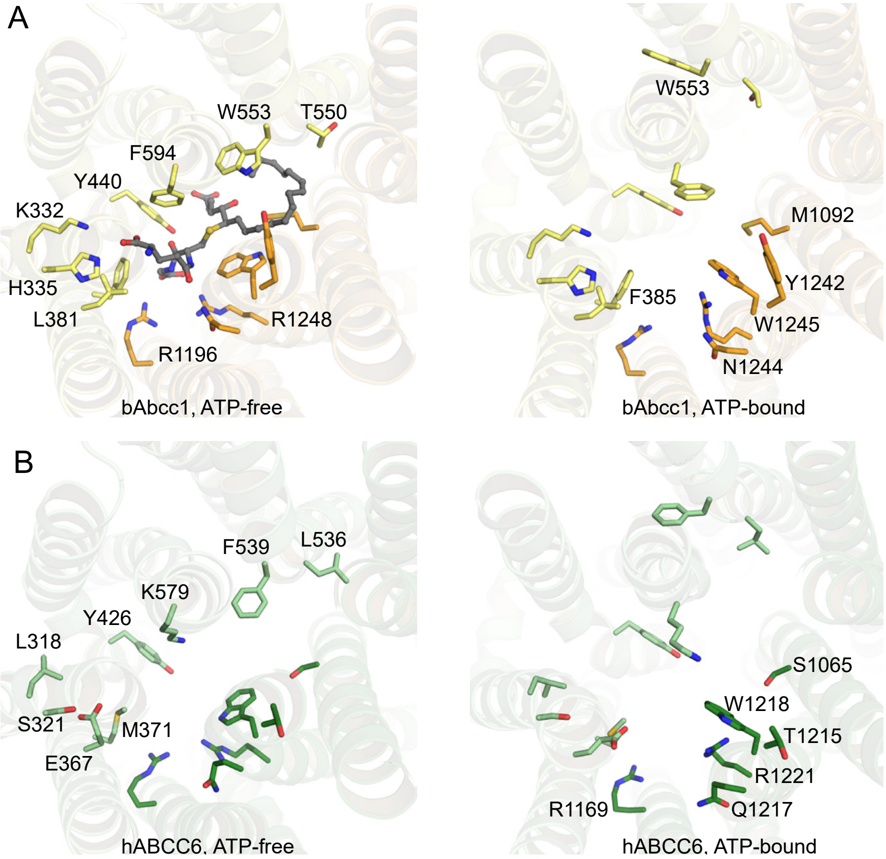

Figure S3. The LTC_4 binding cavity in bAbcc1 and hABCC6. A. Residues in the transmembrane cavity of (left) the ATP-free, inward-facing and (right) ATP-bound, outward-facing experimental structures of bAbcc1 [9, 10]. Residues of TMD1 are shown in yellow sticks, and residues of TMD2 are shown in orange. the LTC4 ligand present in the ATP-free state is shown as gray sticks. B. The corresponding residues in the hABCC6 homology models. Residues of TMD1 are shown in light green sticks, and residues of TMD2 are shown in darker green for the (left) ATP-free, inward-facing and (right) ATP-bound, outward-facing model.

```
MRP1_HUMAN       292   DANEEVEALIV----------KSPQKEWNPSLFKVLYKTFGPYFLMSFFFKAIHDLMMFS    341
MRP2_HUMAN       279   NQSQSQDALVLEDVEKKKKKSGTKKDVPKSWLMKALFKTFYMVLLKSFLLKLVNDIFTFV    338
MRP3_HUMAN       279   NASGE-DEVLL----------GARPRPRKPSFLKALLATFGSSFLISACFKLIQDLLSFI    327
MRP4_HUMAN        72   ------------------------ENDAQKPSLTRAIIKCYWKSYLVLGIFTLIEESAKVI    108
MRP5_HUMAN       163   ---------------------------DAASLRRVVWIFCRTRLILSIVCLMITQLAGFS    195
MRP6_HUMAN       288   --------FL-----------RQEGSQWR-PLLKAIWQVFHSTFLLGTLSLIISDVFRFT    327
                                                      : :.:        :       : :   .

MRP1_HUMAN       342   GPQILKL-LIKFVNDTKAPD----WQGYFYTVLLFVTACLQTLVLHQYFHICFVSGMRIK    396
MRP2_HUMAN       339   SPQLLKL-LISFASDRDTYL----WIGYLCAILLFTAALIQSFCLQCYFQLCFKLGVKVR    393
MRP3_HUMAN       328   NPQLLSI-LIRFISNPMAPS----WWGFLVAGLMFLCSMMQSLILQHYYHYIFVTGVKFR    382
MRP4_HUMAN       109   QPIFLGK-IINYFENYDPMDSVALNTAYAYATVLTFCTLILAILHHLYFYHVQCAGMRLR    167
MRP5_HUMAN       196   GPAFMVKHLLEYTQATESNL----QYSLLLVLGLLLTEIVRSWSLALTWALNYRTGVRLR    251
MRP6_HUMAN       328   VPKLLSL-FLEFIGDPKPPA----WKGYLLAVLMFLSACLQTLFEQQNMYRLKVLQMRLR    382
                        * ::   :: :              .   .  :     : :              ::.:

MRP1_HUMAN       397   TAVIGAVYRKALVITNSARKSSTVGEIVNLMSVDAQRFMDLATYINMIWSAPLQVILALY    456
MRP2_HUMAN       394   TAIMASVYKKALTLSNLARKEYTVGETVNLMSVDAQKLMDVTNFMHMLWSSVLQIVLSIF    453
MRP3_HUMAN       383   TGIMGVIYRKALVITNSVKRASTVGEIVNLMSVDAQRFMDLAPFLNLLWSAPLQIILAIY    442
MRP4_HUMAN       168   VAMCHMIYRKALRLSNMAMGKTTTGQIVNLLSNDVNKFDQVTVFLHFLWAGPLQAIAVTA    227
MRP5_HUMAN       252   GAILTMAFKKILKLKNIK--EKSLGELINICSNDGQRMFEAAAVGSLLAGGPVVAILGMI    309
MRP6_HUMAN       383   SAITGLVYRKVLALSSGSRKASAVGDVVNLVSVDVQRLTESVLYLNGLWLPLVWIVVCFV    442
                        .:    ::* * :..      : *: :*: * * ::: : .     :    :  :

MRP1_HUMAN       457   LLWLNLGPSVLAGVAVMVLMVPVNAVMAMKTKTYQVAHMKSKDNRIKLMNEILNGIKVLK    516
MRP2_HUMAN       454   FLWRELGPSVLAGVGVMVLVIPINAILSTKSKTIQVKNMKNKDKRLKIMNEILSGIKILK    513
MRP3_HUMAN       443   FLWQNLGPSVLAGVAFMVLLIPLNGAVAVKMRAFQVKQMKLKDSRIKLMSEILNGIKVLK    502
MRP4_HUMAN       228   LLWMEIGISCLAGMAVLIILLPLQSCFGKLFSSLRSKTATFTDARIRTMNEVITGIRIIK    287
MRP5_HUMAN       310   YNVIILGPTGFLGSAVFILFYPAMMFASRLTAYFRRKCVAATDERVQKMNEVLTYIKFIK    369
MRP6_HUMAN       443   YLWQLLGPSALTAIAVFLSLLPLNFFISKKRNHHQEEQMRQKDSRARLTSSILRNSKTIK    502
                            :* : : . ..:: . *     .      :      .* * :  ..::   : :*

MRP1_HUMAN       517   LYAWELAFKDKVLAIRQEELKVLKKSAYLSAVGTFTWVCTPFLVALCTFAVYVTIDENNI    576
MRP2_HUMAN       514   YFAWEPSFRDQVQNLRKKELKNLLAFSQLQCVVIFVFQLTPVLVSVVTFSVYVLVDSNNI    573
MRP3_HUMAN       503   LYAWEPSFLKQVEGIRQGELQLLRTAAYLHTTTTFTWMCSPFLVTLITLWVYVYVDPNNV    562
MRP4_HUMAN       288   MYAWEKSFSNLITNLRKKEISKILRSSCLRGMNLASFFSASKIIVFVTFTTYVLLG--SV    345
MRP5_HUMAN       370   MYAWVKAFSQSVQKIREEERRILEKAGYFQSITVGVAPIVVVIASVVTFSVHMTLG--FD    427
MRP6_HUMAN       503   FHGWEGAFLDRVLGIRGQELGALRTSGLLFSVSLVSFQVSTFLVALVVFAVHTLVAE-NA    561
                        ..*  :* . :  :*  *   :   . :             :  . .: .:  :

MRP1_HUMAN       577   LDAQTAFVSLALFNILRFPLN-ILPMVISSIVQASVSLKRLRIFLSHEELEPDSIERRPV    635
MRP2_HUMAN       574   LDAQKAFTSITLFNILRFPLS-MLPMMISSMLQASVSTERLEKYLGGDDLDTSAIRHDCN    632
MRP3_HUMAN       563   LDAEKAFVSVSLFNILRLPLN-MLPQLISNLTQASVSLKRIQQFLSQEELDPQSVERKTI    621
MRP4_HUMAN       346   ITASRVFVAVTLYGAVRLTVTLFFPSAIERVSEAIVSIRRIQTFLLLDEISQRNRQ----    401
MRP5_HUMAN       428   LTAAQAFTVVTVFNSMTFALK-VTPFSVKSLSEASVAVDRFKSLFLMEEVHMIKN-----    481
MRP6_HUMAN       562   MNAEKAFVTLTVLNILNKAQA-FLPFSIHSLVQARVSFDRLVTFLCLEEVDPGVVDSSSS    620
                       : *  .*. ::: . :      . *  :  : :* *:  *:   :  :::

MRP1_HUMAN      1070   GNLVNRFSKELDTVDSMIPEVIKMFMGSLFNVIGACIVILLATPIAAIIIPPLGLIYFFV   1129
MRP2_HUMAN      1078   GRIVNRFAGDISTVDDTLPQSLRSWITCFLGIISTLVMICMATPVFTIIVIPLGIIYVSV   1137
MRP3_HUMAN      1066   GRILNCFSKDIYVVDEVLAPVILMLLNSFFNAISTLVVIMASTPLFTVVILPLAVLYTLV   1125
MRP4_HUMAN       819   GRILNRFSKDIGHLDDLLPLTFLDFIQTLLQVVGVVSVAVAVIPWIAIPLVPLGIIFIFL    878
MRP5_HUMAN       969   GRILNRFSKDMDEVDVRLPFQAEMFIQNVILVFFCVGMIAGVFPWFLVAVGPLVILFSVL   1028
MRP6_HUMAN      1042   GHLLNRFSKETDTVDVDIPDKLRSLLMYAFGLLEVSLVVAVATPLATVAILPLFLLYAGF   1101
                       *.::* *: :   :*  :       :   :  .    :     *   : : ** :::  .

MRP1_HUMAN      1130   QRFYVASSRQLKRLESVSRSPVYSHFNETLLGVSVIRAFEEQERFIHQSDLKVDENQKAY   1189
MRP2_HUMAN      1138   QMFYVSTSRQLRRLDSVTRSPIYSHFSETVSGLPVIRAFEHQQRFLKHNEVRIDTNQKCV   1197
MRP3_HUMAN      1126   QRFYAATSRQLKRLESVSRSPIYSHFSETVTGASVIRAYNRSRDFEIISDTKVDANQRSC   1185
MRP4_HUMAN       879   RRYFLETSRDVKRLESTTRSPVFSHLSSSLQGLWTIRAYKAEERCQELFDAHQDLHSEAW    938
MRP5_HUMAN      1029   HIVSRVLIRELKRLDNITQSPFLSHITSSIQGLATIHAYNKGQEFLHRYQELLDDNQAPF   1088
MRP6_HUMAN      1102   QSLYVVSSCQLRRLESASYSSVCSHMAETFQGSTVVRAFRTQAPFVAQNNARVDESQRIS   1161
                       :        :::**:. : * . **: .:. *  .::*:.         :   *  .

MRP1_HUMAN      1190   YPSIVANRWLAVRLECVGNCIVLFAALFAVISRHSLSAGLVGLSVSYSLQVTTYLNWLVR   1249
MRP2_HUMAN      1198   FSWITSNRWLAIRLELVGNLTVFFSALMMVIYRDTLSGDTVGFVLSNALNITQTLNWLVR   1257
MRP3_HUMAN      1186   YPYIISNRWLSIGVEFVGNCVVLFAALFAVIGRSSLNPGLVGLSVSYSLQVTFALNWMIR   1245
MRP4_HUMAN       939   FLFLTTSRWFAVRLDAICAMFVIIVAFGSLILAKTLDAGQVGLALSYALTLMGMFQWCVR    998
MRP5_HUMAN      1089   FLFTCAMRWLAVRLDLISIALITTTGLMIVLMHGQIPPAYAGLAISYAVQLTGLFQFTVR   1148
MRP6_HUMAN      1162   FPRLVADRWLAANVELLGNGLVFAAATCAVLSKAHLSAGLVGFSVSAALQVTQTLQWVVR   1221
                       :    : **::  :: :    :   .   ::    :    .*: :* :: :   ::: :*
```

Figure S4. Alignment of ABCC1-6 amino acid sequences. Sequences of the human ABCC1 to ABCC6 proteins retrieved from UniprotKB were aligned using Clustal Omega. ABCC6 amino acid residues mutagenized are indicated by colors. Non conserved residues of ABCC6 are highlighted in yellow and residues conserved amongst ABCC1-ABCC6 are highlighted in blue.

References

1. Situ, D.; Haimeur, A.; Conseil, G.; Sparks, K. E.; Zhang, D.; Deeley, R. G.; Cole, S. P., Mutational analysis of ionizable residues proximal to the cytoplasmic interface of membrane spanning domain 3 of the

multidrug resistance protein, MRP1 (ABCC1): glutamate 1204 is important for both the expression and catalytic activity of the transporter. *J Biol Chem* **2004,** *279* (37), 38871-80.

2. Noji, Y.; Inazu, A.; Higashikata, T.; Nohara, A.; Kawashiri, M.; Yu, W.; Todo, Y.; Nozue, T.; Uno, Y.; Hifumi, S.; Mabuchi, H., Identification of two novel missense mutations (p.R1221C and p.R1357W) in the ABCC6 (MRP6) gene in a Japanese patient with pseudoxanthoma elasticum (PXE). *Internal medicine (Tokyo, Japan)* **2004,** *43* (12).

3. Miksch, S.; Lumsden, A.; Guenther, U.; Foernzler, D.; Christen-Zäch, S.; Daugherty, C.; Ramesar, R.; Lebwohl, M.; Hohl, D.; Neldner, K.; Lindpaintner, K.; Richards, R.; Struk, B., Molecular genetics of pseudoxanthoma elasticum: type and frequency of mutations in ABCC6. *Human mutation* **2005,** *26* (3).

4. Pfendner, E.; Vanakker, O. M.; Terry, S.; Vourthis, S.; McAndrew, P. E.; McClain, M. R.; Fratta, S.; Marais, A. S.; Hariri, S.; Coucke, P. J.; Ramsay, M.; Viljoen, D.; Terry, P. F.; De Paepe, A.; Uitto, J.; Bercovitch, L. G., Mutation Detection in the ABCC6 Gene and Genotype-Phenotype Analysis in a Large International Case Series Affected by Pseudoxanthoma Elasticum. *Journal of medical genetics* **2007,** *44* (10).

5. Ren, X.; Furukawa, T.; Aoki, S.; Sumizawa, T.; Haraguchi, M.; Nakajima, Y.; Ikeda, R.; Kobayashi, M.; Akiyama, S., A positively charged amino acid proximal to the C-terminus of TM17 of MRP1 is indispensable for GSH-dependent binding of substrates and for transport of LTC4. *Biochemistry* **2002,** *41* (48).

6. Ryu, S.; Kawabe, T.; Nada, S.; Yamaguchi, A., Identification of basic residues involved in drug export function of human multidrug resistance-associated protein 2. *The Journal of biological chemistry* **2000,** *275* (50).

7. Verschuere, S.; Navassiolava, N.; Martin, L.; Nevalainen, P.; Coucke, P.; Vanakker, O., Reassessment of causality of ABCC6 missense variants associated with pseudoxanthoma elasticum based on Sherloc. *Genetics in medicine : official journal of the American College of Medical Genetics* **2020**.

8. Nitschke, Y.; Baujat, G.; Botschen, U.; Wittkampf, T.; du Moulin, M.; Stella, J.; Le Merrer, M.; Guest, G.; Lambot, K.; Tazarourte-Pinturier, M. F.; Chassaing, N.; Roche, O.; Feenstra, I.; Loechner, K.; Deshpande, C.; Garber, S. J.; Chikarmane, R.; Steinmann, B.; Shahinyan, T.; Martorell, L.; Davies, J.; Smith, W. E.; Kahler, S. G.; McCulloch, M.; Wraige, E.; Loidi, L.; Höhne, W.; Martin, L.; Hadj-Rabia, S.; Terkeltaub, R.; Rutsch, F., Generalized Arterial Calcification of Infancy and Pseudoxanthoma Elasticum Can Be Caused by Mutations in Either ENPP1 or ABCC6. *American journal of human genetics* **2012,** *90* (1).

9. Johnson, Z. L.; Chen, J., Structural Basis of Substrate Recognition by the Multidrug Resistance Protein MRP1. *Cell* **2017,** *168* (6), 1075-1085 e9.

10. Johnson, Z. L.; Chen, J., ATP Binding Enables Substrate Release from Multidrug Resistance Protein 1. *Cell* **2018,** *172* (1-2), 81-89 e10.
